# The effect of heat acclimation or acclimatisation on exercise performance and capacity in the heat: preliminary meta-analysis data

**DOI:** 10.1186/2046-7648-4-S1-A122

**Published:** 2015-09-14

**Authors:** Tom Reeve, Gary J Hodges, Stephen S Cheung, Christopher J Tyler

**Affiliations:** 1 Department of Sport and Exercise Science, University of Roehampton, London, UK; 2 Environmental Ergonomics Laboratory, Department of Kinesiology, Brock University, St. Catharines, Canada

## Introduction

Exercise performance and capacity are impaired in hot and humid compared to temperate conditions [1], [3] and so researchers, coaches and athletes are interested in strategies to attenuate this impairment. Heat acclimation (or acclimatisation) (HA) is one approach that may reduce the thermal strain of exercising in hot conditions and benefit exercise performance [1], [2]. This meta-analysis quantified the magnitude of effect that HA has on exercise performance and capacity in the heat, and tested whether the magnitude of effect is related to the volume or intensity of heat stress experienced.

## Methods

The PubMed database was searched (09/01/15) using the first-order search terms acclimation, acclimatization, acclimatisation and adaptation and second-order search terms heat, exercise, performance, capacity and training. Using the four-stage process identified in the PRISMA statement the initial number of results (9,369) was reduced to 93. Data (N, mean, SD) were extracted from these articles in duplicate or triplicate. A subset of the data (n = 29 manuscripts) are presented here; manuscripts were included if exercise performance (Time to complete a fixed amount of work: E.g., time trial) and/or capacity (Open ended tests: E.g., Maximal aerobic power, time to exhaustion at a fixed workload), were measured and reported. All HA protocols regardless of duration, frequency, ambient conditions or exercise modality were used. Hedge's g (± 95 % CI) were calculated and correlation analyses were performed between the effect size and total HA time (HA_time_), and HA temperature (HA_temp_).

## Results

The 29 manuscripts reviewed used a mean (± SD) of 9.8 ± 4.0 (range: 4 - 24) HA sessions separated by 0.2 ± 0.4 (0 - 2) days. Total HA_time _was 1055 ± 746 min (190 - 3,120), and the HA_temp _and HA_humidity _were 39.9 ± 5.9 °C (30 - 49) and 34 ± 17 % rh (14 - 87), respectively.

## Conclusion

HA is an effective way to improve both exercise capacity and performance in the heat. The magnitude of the effect appears to be independent of either HA_time _or HA_temp _for capacity, and independent of HA_temp _for performance. However, the magnitude of benefit on exercise performance may be dependent upon HA_time_.

**Table 1:** The effect of HA on exercise performance and capacity (n = 7 walking, n = 8 running, n = 13 cycling, n = 1 rowing)

	Manuscripts	Groups	N	Hedges *g *(± 95% CI)	Mean Δ	HA_time_	HA_temp_
Capacity	15	19	209	0.60 (0.40, 0.81)	+ 21 %	*r *= 0.22^NS^	*r *= 0.11^NS^

Performance	10	22	198	0.58 (0.37, 0.79)	+ 5.3 %	*r *= 0.67**	*r *= 0.06^NS^

^** ^= *P *<0.01; ^NS ^= *P *= 0.37 - 0.80
